# Incidence of Surgical Site Infection After Elective and Emergency Abdominal Surgeries: A Prospective Comparative Study

**DOI:** 10.7759/cureus.115242

**Published:** 2026-08-26

**Authors:** Pratik Shaparia, Hemal Dholakia, Harmika Parmar

**Affiliations:** 1 General Surgery, Smt. B. K. Shah Medical Institute and Research Centre, Sumandeep Vidyapeeth (Deemed to be University), Vadodara, IND; 2 Pharmacology, Dr N.D. Desai Medical College &amp; Hospital, Dharmsinh Desai University, Nadiad, IND; 3 Microbiology, Dr. M.P. Shah Government Medical College, Saurashtra University, Jamnagar, IND

**Keywords:** abdominal surgery, antibiotic sensitivity, elective surgery, emergency surgery, escherichia coli, infection surveillance, postoperative outcomes, postoperative wound infection, surgical morbidity, surgical site infection

## Abstract

Background

Surgical site infection (SSI) is a common postoperative complication associated with increased morbidity, mortality, prolonged hospitalization, and increased healthcare costs. This study assessed the incidence, microbiological profile, factors associated with SSI, and postoperative outcomes among patients undergoing elective and emergency abdominal surgeries.

Methods

A prospective comparative observational study was conducted among 100 adult patients undergoing abdominal surgery at a tertiary care hospital, comprising 50 patients undergoing elective surgery and 50 undergoing emergency surgery. Participants were followed for 30 days postoperatively for the development of SSI. Demographic and clinical characteristics, microbiological findings, antimicrobial susceptibility patterns, response to antibiotic therapy, and clinical outcomes were recorded. Associations between selected demographic and clinical characteristics and SSI were assessed using the chi-square test or Fisher's exact test, as appropriate. A p-value of <0.05 was considered statistically significant.

Results

SSI occurred in 30 (30.0%) patients overall, including 18 (36.0%) patients undergoing emergency surgery and 12 (24.0%) undergoing elective surgery. *Escherichia coli* was the most frequently isolated organism (14; 46.7%), followed by *Staphylococcus aureus* (10; 33.3%). Among the patients who developed SSI, 24 (80.0%) showed sensitivity to the tested antibiotics, whereas 6 (20.0%) showed resistance. A clinical response to broad-spectrum antibiotic therapy was observed in 17 (56.7%) patients, whereas 13 (43.3%) did not respond adequately. Overall, 82 (82.0%) patients were discharged, and 18 (18.0%) died during the study period. No statistically significant association was observed between SSI and surgery type, Centers for Disease Control and Prevention (CDC) wound classification, age group, past history, or comorbidities (p > 0.05).

Conclusion

SSI occurred in both elective and emergency abdominal surgery groups, with a numerically higher incidence in the emergency group. *Escherichia coli* was the predominant organism isolated from SSI cases. No significant associations were observed between SSI and the demographic, clinical, and selected surgical characteristics assessed. Further studies incorporating comprehensive perioperative factors and multivariable analysis are warranted to identify independent predictors of SSI.

## Introduction

Surgical site infections (SSIs) are among the most common healthcare-associated infections and remain a significant cause of postoperative morbidity and mortality worldwide [1,2]. They contribute substantially to prolonged hospital stays, increased healthcare expenditure, delayed wound healing, and reduced quality of life. SSIs may also necessitate additional surgical interventions, intensive antimicrobial therapy, and hospital readmissions, thereby placing a considerable burden on healthcare systems. Furthermore, the emergence of antimicrobial resistance has made the prevention and appropriate management of SSIs an important public health priority.

The incidence of SSI varies across geographical regions, healthcare settings, and types of surgical procedures. Approximately 2% of hospitalized patients are estimated to develop SSIs, although the actual burden may be higher because some infections occur after discharge and may remain underreported [3,4]. In abdominal surgery, reported SSI rates range from 3% to 20%, with higher rates observed among high-risk patients and those undergoing emergency procedures [5,6]. SSI is associated with increased morbidity, delayed recovery, prolonged disability, and a substantial economic burden for both patients and healthcare institutions.

According to the Centers for Disease Control and Prevention (CDC), an SSI is an infection occurring within 30 days following an operative procedure and involving the incision or operative site [7-9]. Surgical wounds are classified as clean, clean-contaminated, contaminated, or dirty according to the degree of microbial contamination during surgery. The risk of SSI generally increases with increasing wound contamination. The development of SSI is multifactorial and influenced by the interaction between microbial contamination, bacterial virulence, local wound conditions, host factors, and perioperative factors.

In developing countries such as India, SSIs remain a major challenge because of variations in healthcare infrastructure, infection prevention practices, and patient- and procedure-related factors. Emergency abdominal surgeries may have a higher risk of SSI because of limited opportunities for preoperative optimization, increased wound contamination, and the underlying conditions necessitating urgent surgery [10]. Several factors, including advanced age, diabetes mellitus, anemia, smoking, obesity, malnutrition, wound contamination, and prolonged operative duration, have been reported to be associated with postoperative wound infection [11,12]. Evaluation of these factors may help identify patients at increased risk and support appropriate infection-prevention strategies.

The microbiological profile of SSIs has also evolved over time, with increasing concern regarding antimicrobial-resistant organisms, including *Staphylococcus aureus* and *Escherichia coli* [13]. Knowledge of local microbial patterns and antimicrobial susceptibility is important for guiding empirical therapy, supporting antibiotic stewardship, and reducing treatment failures. Despite advances in surgical techniques and infection-prevention measures, SSIs continue to adversely affect postoperative recovery and remain an important cause of morbidity following abdominal surgery.

Therefore, the present study was undertaken to determine the incidence of postoperative SSI among patients undergoing elective and emergency abdominal surgeries, compare SSI occurrence between the two groups, assess selected demographic and clinical factors associated with SSI, describe the microbiological profile and antimicrobial susceptibility of SSI isolates, and evaluate postoperative clinical outcomes.

## Materials and methods

Study design and setting

A prospective comparative observational study was conducted in the Department of General Surgery, Dhiraj General Hospital, Piparia, Vadodara, Gujarat, India, over a period of 18 months. The study evaluated the incidence of postoperative SSI, selected patient- and procedure-related factors associated with SSI, the microbiological profile and antimicrobial susceptibility patterns of SSI isolates, and postoperative outcomes among patients undergoing elective and emergency abdominal surgeries.

Study population and sampling

The study population comprised adult patients admitted to the Department of General Surgery who underwent abdominal surgical procedures during the study period. Consecutive sampling was used, and eligible patients were enrolled until 50 patients undergoing elective surgery and 50 patients undergoing emergency surgery had been included. Thus, a total of 100 participants were included, with equal allocation between the two surgical groups.

Because the study was conducted within a predefined study period and was subject to feasibility and recruitment constraints, the final sample of 100 participants was considered a pragmatic feasibility sample rather than a formally powered comparative sample. The sample size was therefore not based on the single-proportion formula, and no claim of adequate statistical power to detect a difference in SSI incidence between elective and emergency surgery groups was made.

Eligibility criteria

Inclusion Criteria

Patients aged ≥18 years who underwent abdominal surgery at the study center were eligible for inclusion. Patients were consecutively enrolled into two predefined groups: elective abdominal surgery and emergency abdominal surgery. Written informed consent was obtained from all participants.

Exclusion Criteria

Patients with a documented history of previous SSI, those who underwent reoperation for indications other than management of SSI, those who left the hospital against medical advice, those who failed to complete the 30-day postoperative follow-up period, and those unwilling to participate were excluded from the study.

Study tools and data collection

Data were collected using a predesigned and pretested case record form developed for the study. The case record form captured demographic characteristics, clinical history, comorbid conditions, lifestyle factors, laboratory parameters, operative details, wound classification, postoperative outcomes, and microbiological findings.

All participants underwent detailed history-taking and comprehensive physical examination. Baseline investigations included complete blood count, blood glucose estimation, renal function tests, and other investigations as clinically indicated. Additional diagnostic evaluations were performed according to institutional protocols.

Information regarding the type of surgery (elective or emergency), indication for surgery, CDC wound classification, and selected perioperative management details was recorded. Patients were monitored during their hospital stay and followed for 30 days postoperatively for the occurrence of SSI.

SSI was defined according to the CDC criteria as an infection occurring within 30 days after surgery involving the incision or deep tissues. SSIs were categorized as superficial incisional SSI, deep incisional SSI, and organ/space SSI [14].

SSI was identified based on clinical findings such as purulent discharge, localized swelling, erythema, pain, tenderness, wound dehiscence, or surgeon diagnosis. When SSI was suspected, wound specimens were collected under aseptic conditions and sent for microbiological culture and antimicrobial susceptibility testing.

Operative characteristics

Surgical wounds were classified into four categories - clean, clean-contaminated, contaminated, and dirty/infected - based on the degree of microbial contamination at the time of surgery, as defined by the CDC surgical wound classification system [15].

The underlying diagnoses included perforation-related conditions, intestinal obstruction, appendicitis-related conditions, malignancy, gynecological abdominal conditions, hernia, hepatobiliary and splenic conditions, trauma, pancreatic conditions, and other abdominal conditions.

Outcome measures

The primary outcome measure was the incidence of postoperative SSI within 30 days of surgery. Secondary outcomes included identification of associated patient- and procedure-related factors, characterization of microorganisms causing SSI, duration of hospital stay, postoperative morbidity, and mortality.

Handling of missing data

No missing data were observed in the study. All 100 enrolled participants completed the 30-day follow-up and were included in the final analysis.

Ethical considerations

The study protocol was reviewed and approved by the Institutional Ethics Committee of Sumandeep Vidyapeeth (approval number: SVIEC/ON/MEDI/BNPG23/AUG/24/50 dated 29 August 2024). Written informed consent was obtained from all participants prior to enrollment. Confidentiality and anonymity of participant information were maintained throughout the study, and all procedures were conducted in accordance with the Declaration of Helsinki.

Statistical analysis

Data were entered into Microsoft Excel 2016 (Microsoft Corporation, Redmond, WA, USA) and analyzed using IBM SPSS Statistics for Windows, Version 26.0 (IBM Corp., Armonk, NY, USA). Categorical variables were summarized as frequencies and percentages. The incidence of SSI was calculated as the proportion of participants who developed SSI during the 30-day postoperative follow-up. Associations between selected demographic and clinical characteristics and SSI status were assessed using the chi-square test or Fisher’s exact test, as appropriate. A p-value of <0.05 was considered statistically significant.

Given the relatively small sample size (n = 100) and the limited number of SSI events (n = 30), multivariable logistic regression was not performed because the available number of outcome events was insufficient for a reliable model incorporating the multiple potential perioperative and patient-related variables. Therefore, analyses of factors associated with SSI were restricted to univariable comparisons, and the findings were interpreted as exploratory associations rather than independent predictors.

## Results

A total of 100 patients were included in the study, with 50 undergoing elective surgery and 50 undergoing emergency surgery. Among elective surgery patients, the largest proportion belonged to the age group of 41-50 years (15; 30.0%), whereas among emergency surgery patients, the highest proportion was observed among those aged >60 years (14; 28.0%). Males constituted 29 (58.0%) of elective surgery patients and 37 (74.0%) of emergency surgery patients. Previous surgery was reported by 11 (22.0%) patients in the elective group and 9 (18.0%) in the emergency group. Most participants had no significant past history, 30 (60.0%) and 34 (68.0%), respectively, and no comorbidities, 44 (88.0%) and 39 (78.0%), respectively. No statistically significant differences were observed between the elective and emergency surgery groups with respect to age group (χ² = 7.03, p = 0.216), gender (χ² = 2.85, p = 0.091), past history (Fisher’s exact test, p = 0.158), or comorbidities (Fisher’s exact test, p = 0.658) (Table 1).

**Table 1:** Baseline sociodemographic and clinical characteristics of participants according to type of surgery (N = 100). Data are presented as n (%). Percentages are calculated within each surgery group. Pearson’s chi-square test was used for age group and gender, while Fisher’s exact test was used for past history and comorbidities because of sparse cell frequencies. A p-value of <0.05 was considered statistically significant.

Variable	Elective Surgery (n=50)	Emergency Surgery (n=50)	Test Statistic	p-Value
Age group (years)				
<20	2 (4.0%)	3 (6.0%)	χ² = 7.03	0.218
21–30	8 (16.0%)	7 (14.0%)		
31–40	7 (14.0%)	10 (20.0%)		
41–50	15 (30.0%)	6 (12.0%)		
51–60	11 (22.0%)	10 (20.0%)		
>60	7 (14.0%)	14 (28.0%)		
Gender				
Male	29 (58.0%)	37 (74.0%)	χ² = 2.85	0.091
Female	21 (42.0%)	13 (26.0%)		
Past history				
Previous surgery	11 (22.0%)	9 (18.0%)	Fisher’s exact test	0.158
Trauma	0 (0.0%)	3 (6.0%)		
Addiction habits	9 (18.0%)	4 (8.0%)		
None	30 (60.0%)	34 (68.0%)		
Comorbidities				
Abdominal Koch's	1 (2.0%)	1 (2.0%)	Fisher’s exact test	0.658
Malignancy	3 (6.0%)	2 (4.0%)		
Asthma	0 (0.0%)	1 (2.0%)		
Hypertension	1 (2.0%)	2 (4.0%)		
Diabetes mellitus	1 (2.0%)	3 (6.0%)		
Pancreatitis	0 (0.0%)	2 (4.0%)		
None	44 (88.0%)	39 (78.0%)		

Perforation-related conditions were the most common (30; 30.0%), all occurring in the emergency group. Intestinal obstruction accounted for 11 (11.0%) cases, appendicitis-related conditions for 9 (9.0%) cases, and malignancy-related conditions for 10 (10.0%) cases. Gynecological conditions accounted for 14 (14.0%) cases, all elective. Hernia-related, hepatobiliary, trauma-related, splenic, and pancreatic conditions accounted for six (6.0%), six (6.0%), five (5.0%), four (4.0%), and three (3.0%) cases, respectively, while other diagnoses accounted for two (2.0%) cases (Table 2).

**Table 2:** Distribution of abdominal surgical diagnoses according to type of surgery (N = 100) Data are presented as n (%). Percentages represent the proportion of the total study population (N = 100). Diagnoses were grouped according to the underlying clinical diagnosis documented for the surgical admission.

Diagnosis Category	Elective, n (%)	Emergency, n (%)	Total, n (%)
Perforation-related conditions	0 (0.0%)	30 (30.0%)	30 (30.0%)
Gynecological abdominal conditions	14 (14.0%)	0 (0.0%)	14 (14.0%)
Intestinal obstruction	5 (5.0%)	6 (6.0%)	11 (11.0%)
Malignancy-related conditions	6 (6.0%)	4 (4.0%)	10 (10.0%)
Appendicitis-related conditions	1 (1.0%)	8 (8.0%)	9 (9.0%)
Hernia-related conditions	4 (4.0%)	2 (2.0%)	6 (6.0%)
Hepatobiliary conditions	3 (3.0%)	3 (3.0%)	6 (6.0%)
Trauma-related conditions	1 (1.0%)	4 (4.0%)	5 (5.0%)
Splenic conditions	2 (2.0%)	2 (2.0%)	4 (4.0%)
Pancreatic conditions	1 (1.0%)	2 (2.0%)	3 (3.0%)
Other conditions	1 (1.0%)	1 (1.0%)	2 (2.0%)
Total	50 (50.0%)	50 (50.0%)	100 (100.0%)

Surgical wounds were classified according to the CDC surgical wound classification system. Among elective procedures, clean-contaminated wounds were predominant (36; 72.0%), followed by clean wounds (11; 22.0%), dirty/infected wounds (2; 4.0%), and contaminated wounds (1; 2.0%). Among emergency procedures, dirty/infected wounds were predominant (40; 80.0%), followed by clean-contaminated (5; 10.0%), contaminated (4; 8.0%), and clean wounds (1; 2.0%). The distribution of wound classification differed significantly between elective and emergency surgeries (Fisher's exact test, p < 0.001) (Table 3).

**Table 3:** Distribution of surgical wounds according to CDC wound classification by type of surgery (N = 100) Data are presented as n (%). Percentages are calculated within each surgery group. Surgical wounds were classified as clean, clean-contaminated, contaminated, or dirty/infected according to the CDC surgical wound classification system. Fisher’s exact test was used because of small cell frequencies in the contingency table and demonstrated a statistically significant association between surgery type and wound classification (p < 0.001). CDC, Centers for Disease Control and Prevention

CDC Wound Classification	Elective, n (%)	Emergency, n (%)	Total, n (%)
Clean	11 (22.0)	1 (2.0)	12 (12.0)
Clean-contaminated	36 (72.0)	5 (10.0)	41 (41.0)
Contaminated	1 (2.0)	4 (8.0)	5 (5.0)
Dirty/infected	2 (4.0)	40 (80.0)	42 (42.0)
Total	50 (100.0)	50 (100.0)	100 (100.0)

**Figure 1:**
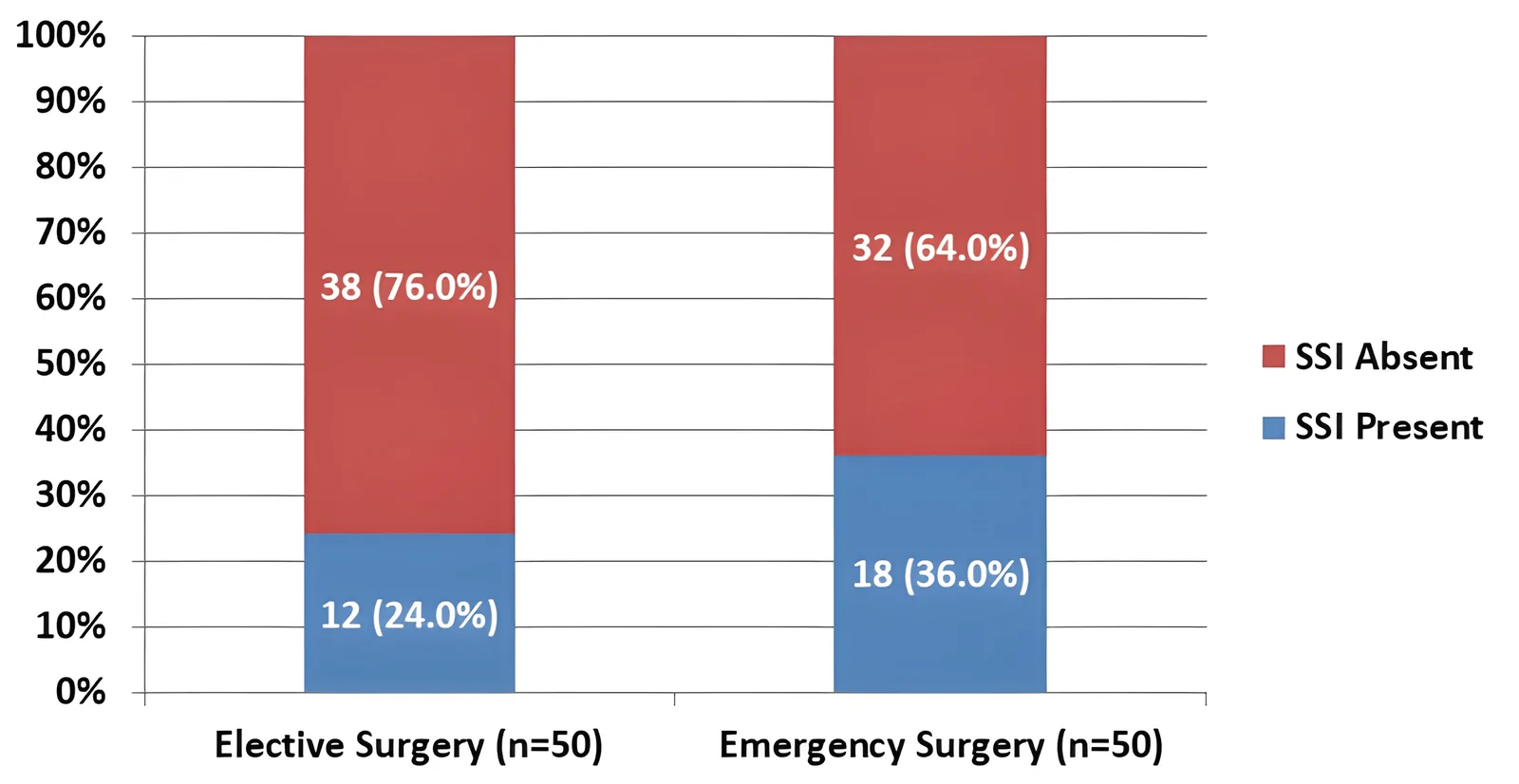
Comparison of SSI incidence between elective and emergency surgery groups (N = 100). The stacked bar chart illustrates the proportion of patients with and without SSI in the elective surgery (n = 50) and emergency surgery (n = 50) groups. Percentages are calculated within each surgery group. SSI, surgical site infection

SSI occurred in 30 (30.0%) patients overall, including 18 (36.0%) patients undergoing emergency surgery and 12 (24.0%) undergoing elective surgery (Figure 1).

**Figure 2:**
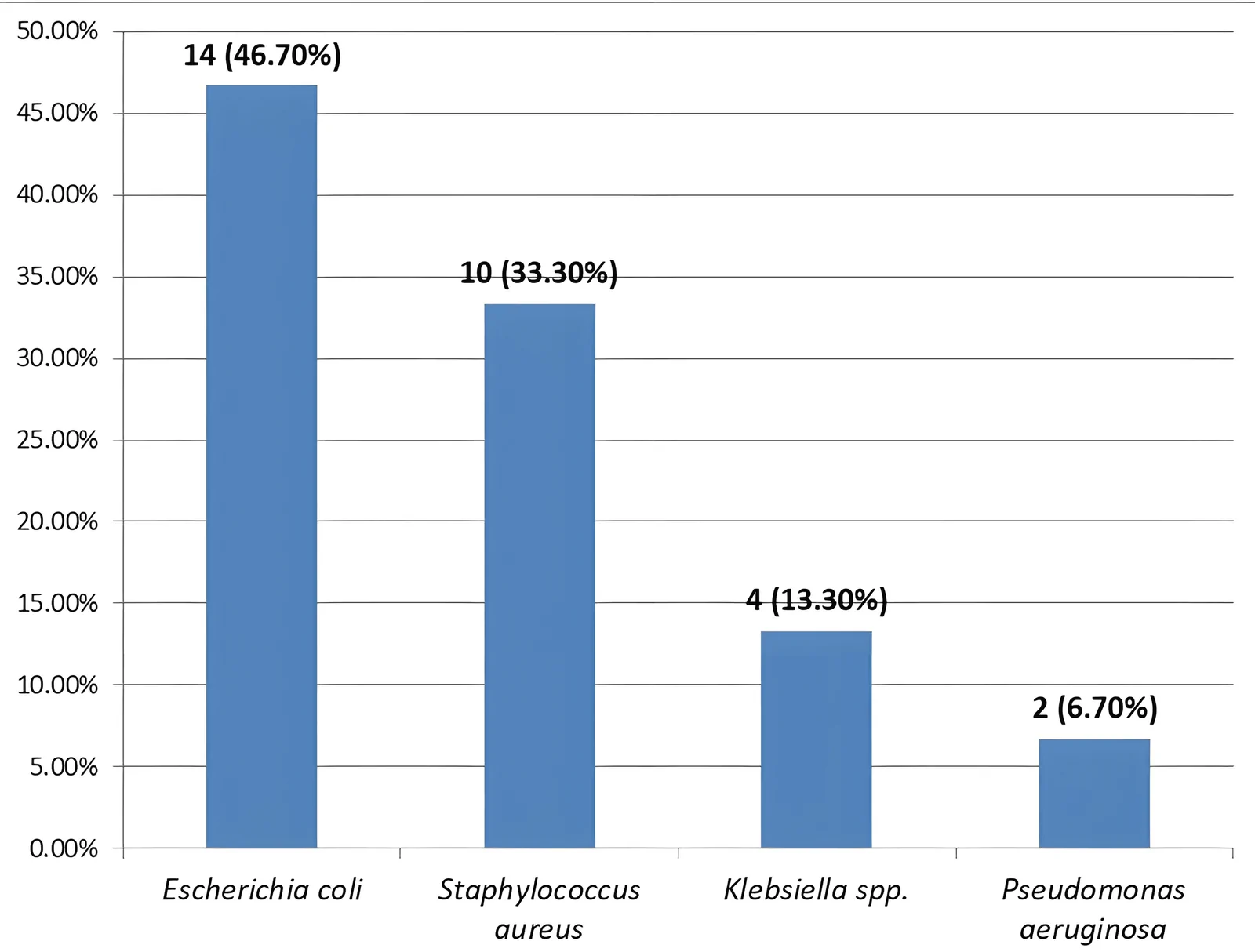
Distribution of bacterial isolates among patients with SSI (n = 30). The figure shows the frequency and percentage of bacterial pathogens isolated from  wound cultures. Percentages are calculated among culture-positive SSI cases (n = 30). SSI, surgical site infection

Microbiological analysis of SSI cases showed that *Escherichia coli* was the most frequently isolated organism, accounting for 14 (46.7%) isolates, followed by *Staphylococcus aureus *(10; 33.3%), *Klebsiella *spp. (4; 13.3%), and *Pseudomonas aeruginosa *(2; 6.7%) (Figure 2).

Among the 30 patients who developed SSI, 17 (56.7%) responded to broad-spectrum antibiotics, including 7 (63.6%) elective and 10 (52.6%) emergency surgery patients, while 13 (43.3%) did not respond, including 4 (36.4%) elective and 9 (47.4%) emergency surgery patients. Among the 30 patients with SSI, antimicrobial susceptibility testing showed that 24 (80.0%) isolates were susceptible to the tested antibiotics, including 8 (72.7%) from the elective surgery group and 16 (84.2%) from the emergency surgery group. Six (20.0%) isolates were resistant, including three (27.3%) from the elective surgery group and three (15.8%) from the emergency surgery group. Overall, 82 (82.0%) patients were discharged, and 18 (18.0%) died; mortality was 12.0% among elective surgery patients and 24.0% among emergency surgery patients (Table 4).

**Table 4:** Clinical outcomes, antimicrobial susceptibility pattern, and response to broad-spectrum antibiotics among patients (N = 100) Data are presented as n (%). Percentages for response to broad-spectrum antibiotics and antimicrobial susceptibility were calculated within each surgery group among patients who developed SSI (elective, n = 11; emergency, n = 19). Percentages for overall clinical outcomes were calculated within each surgery group using all study participants (n = 50 per group). Antimicrobial susceptibility results were available only for 30 SSI isolates. SSI, surgical site infection

Variable	Elective Surgery, n (%)	Emergency Surgery, n (%)	Total, n (%)
Response to broad-spectrum antibiotics among SSI cases (n = 30)			
Responded	7 (63.6%)	10 (52.6%)	17 (56.7%)
Did not respond	4 (36.4%)	9 (47.4%)	13 (43.3%)
Antimicrobial susceptibility among SSI isolates (n = 30)			
Susceptible	8 (72.7%)	16 (84.2%)	24 (80.0%)
Resistant	3 (27.3%)	3 (15.8%)	6 (20.0%)
Overall clinical outcome (n = 100)			
Discharged	44 (88.0%)	38 (76.0%)	82 (82.0%)
Death	6 (12.0%)	12 (24.0%)	18 (18.0%)

Univariable analysis of selected demographic and clinical characteristics showed no statistically significant association between SSI and surgery type (χ² = 3.05, p = 0.081), CDC wound classification (Fisher’s exact test, p = 0.639), age group (χ² = 6.86, p = 0.231), past history (Fisher’s exact test, p = 0.549), or comorbidities (Fisher’s exact test, p = 0.819). Among patients with SSI, 19 (63.3%) underwent emergency surgery and 11 (36.7%) underwent elective surgery. Dirty/infected wounds accounted for 15 (50.0%) SSI cases, followed by clean-contaminated wounds (10; 33.3%), clean wounds (3; 10.0%), and contaminated wounds (2; 6.7%). Descriptively, the largest proportion of SSI cases was observed among patients aged 51-60 years (10; 33.3%), those with addiction habits (11; 36.7%), and those with malignancy (5; 16.7%) or diabetes mellitus (4; 13.3%) (Table 5).

**Table 5:** Association of demographic and clinical factors with SSI among study participants (n = 100) Data are presented as n (%). Percentages were calculated column-wise using the total number of participants with SSI (n = 30) and without SSI (n = 70) as denominators. Pearson’s chi-square (χ²) test was used for variables with adequate expected cell frequencies, while Fisher’s exact test was used when expected cell frequencies were small, i.e., less than 5. A p-value of <0.05 was considered statistically significant. The analysis represents univariable associations and does not establish independent risk factors. CDC, Centers for Disease Control and Prevention; SSI, surgical site infection

Variable	SSI Present, n (%)	SSI Absent, n (%)	Test Statistic	p-Value
Surgery type				
Elective	11 (36.7)	39 (55.7)	χ² = 3.05	0.081
Emergency	19 (63.3)	31 (44.3)		
CDC wound classification				
Clean	3 (10.0)	9 (12.9)	Fisher's exact test	0.639
Clean-contaminated	10 (33.3)	31 (44.3)		
Contaminated	2 (6.7)	3 (4.3)		
Dirty/infected	15 (50.0)	27 (38.6)		
Age group (years)				
<20	1 (3.3)	4 (5.7)	χ² = 6.86	0.231
21–30	2 (6.7)	13 (18.6)		
31–40	6 (20.0)	11 (15.7)		
41–50	4 (13.3)	17 (24.3)		
51–60	10 (33.3)	11 (15.7)		
>60	7 (23.3)	14 (20.0)		
Past history				
Previous surgery	4 (13.3)	20 (28.6)	Fisher's exact test	0.549
Trauma	2 (6.7)	1 (1.4)		
Addiction habits	11 (36.7)	2 (2.9)		
None	13 (43.3)	51 (72.9)		
Comorbidities				
Abdominal Koch's	1 (3.3)	1 (1.4)	Fisher's exact test	0.819
Malignancy	5 (16.7)	0 (0.0)		
Asthma	0 (0.0)	1 (1.4)		
Hypertension	1 (3.3)	2 (2.9)		
Diabetes mellitus	4 (13.3)	0 (0.0)		
Pancreatitis	0 (0.0)	2 (2.9)		
None	19 (63.3)	64 (91.4)		

## Discussion

The present study found SSI in 30 (30.0%) patients, occurring in 12 (24.0%) elective and 18 (36.0%) emergency abdominal surgeries. Mawalla et al. [10] reported an SSI rate of 26.0% among patients undergoing major surgical procedures in Tanzania, while Alkaaki et al. [16] documented an incidence of 32.0% following abdominal surgery in a tertiary care hospital. Differences in reported SSI rates may reflect variations in patient populations, surgical case mix, wound contamination, and infection-prevention practices. Alkaaki et al. [16] identified emergency surgery as an independent predictor of SSI; however, the difference in SSI occurrence between elective and emergency surgery was not statistically significant in the present study (p = 0.081).

The baseline characteristics of the two groups were broadly similar. Patients aged 41-50 years were most common in the elective group (15; 30.0%), while those aged >60 years were most common in the emergency group (14; 28.0%). No significant differences were observed between the groups in age distribution (χ² = 7.03, p = 0.216), gender (χ² = 2.85, p = 0.091), past history (Fisher’s exact test, p = 0.158), or comorbidities (Fisher’s exact test, p = 0.658).

Regarding factors associated with SSI, age group was not significantly associated with SSI (χ² = 6.86, p = 0.231). Patients aged 51-60 years constituted the largest proportion of SSI cases, but this was a descriptive finding. Anderson [17] identified increasing age among patient-related factors considered in relation to SSI, while Ban et al. [11] and Berríos-Torres et al. [9] reported age as a relevant factor in SSI risk. Differences between these findings and the present study may reflect differences in study populations and sample sizes.

Past history and comorbidities were also not significantly associated with SSI (p = 0.549 and p = 0.819, respectively). Addiction habits, malignancy, and diabetes mellitus were observed among patients with SSI, but these findings were descriptive and did not demonstrate statistically significant associations. Diabetes mellitus, malignancy, and other host-related factors have nevertheless been recognized as relevant considerations in SSI prevention and surveillance [9,11].

CDC wound classification differed between elective and emergency procedures, with clean-contaminated wounds predominating among elective procedures and dirty/infected wounds predominating among emergency procedures. However, wound classification was not significantly associated with SSI in the present study (Fisher’s exact test, p = 0.639). In contrast, Jahangir et al. [18] identified higher wound class as one of the more consistently reported factors associated with SSI following abdominal surgery. This difference may reflect variations in sample size and wound-class distribution.

The predominant organism isolated from SSI cases was *Escherichia coli*, followed by *Staphylococcus aureus*, *Klebsiella *spp., and *Pseudomonas aeruginosa*. This pattern differed from that reported by Narula et al. [19], in which *Staphylococcus aureus* followed by *Klebsiella pneumoniae* were the predominant organisms in postoperative wound infections. Owens and Stoessel [5] similarly identified gram-negative enteric organisms and *Staphylococcus aureus* as important pathogens in abdominal SSIs. Differences in bacterial distribution may reflect variations in surgical case mix and local microbiological patterns.

Among the 30 patients with SSI, 24 (80.0%) demonstrated susceptibility to the tested antibiotics, whereas 6 (20.0%) demonstrated resistance. A clinical response to broad-spectrum antibiotic therapy was observed in 17 (56.7%) patients, while 13 (43.3%) did not respond adequately. These represent distinct outcomes and should not be considered interchangeable. Alkaaki et al. [16] reported that only 23% of cultured organisms were susceptible to the prophylactic antibiotic administered preoperatively, whereas Narula et al. [19] reported resistance patterns that varied according to the bacterial isolate and antimicrobial agent. Differences in susceptibility findings may therefore reflect differences in organisms, antimicrobial agents tested, and local resistance patterns. Clinical response should consequently be considered alongside microbiological findings and clinical management.

Overall mortality was 12.0% among elective surgery patients and 24.0% among emergency surgery patients. The present study did not determine whether individual deaths were attributable to SSI; therefore, these findings represent overall postoperative clinical outcomes and should not be interpreted as SSI-related mortality. Smyth et al. [20] reported that SSIs constituted 14.5% of healthcare-associated infections in a large multicountry prevalence survey, illustrating the contribution of SSI to the overall healthcare-associated infection burden.

The findings should be interpreted in the context of the multifactorial nature of SSI. Jahangir et al. [18] reported a pooled SSI incidence of 10.6% following abdominal surgery and identified operative duration and higher wound class as consistently reported factors associated with SSI. Alkaaki et al. [16] similarly reported associations with emergency surgery, open surgical approach, prolonged operative duration, and male sex. In the present study, surgery type, wound classification, age, past history, and comorbidities were examined, but none showed a statistically significant association with SSI. The differences from previous studies may partly reflect the relatively small sample size and the limited number of variables assessed.

The present analysis was based on univariable comparisons and therefore does not establish independent predictors of SSI. Important perioperative variables, including operative duration, surgical approach, ASA grade, nutritional status, timing of antibiotic prophylaxis, blood transfusion, drain placement, and detailed intraoperative contamination, were not comprehensively assessed. Consequently, the absence of statistically significant associations in the present study should not be interpreted as evidence that these factors are unrelated to SSI. Future studies with larger sample sizes and comprehensive assessment of perioperative variables, preferably using multivariable analysis, would provide a more complete assessment of factors associated with SSI.

Limitations

The present study has several limitations. First, it was conducted at a single tertiary care center with a relatively small sample size, which may limit the generalizability of the findings. Second, the observational design allows assessment of associations but does not establish causal relationships. Third, the study had only 30 SSI events, limiting the reliability of multivariable modelling with multiple potential predictors; consequently, the analysis was restricted to univariable comparisons and independent predictors of SSI could not be established. Fourth, although CDC wound classification was assessed, several potentially important perioperative factors, including operative duration, surgical approach, ASA grade, nutritional status, timing of antibiotic prophylaxis, blood transfusion, drain placement, and detailed intraoperative contamination, were not comprehensively evaluated. Finally, the microbiological profile and antimicrobial susceptibility patterns may reflect local epidemiological characteristics and may not be generalizable to other healthcare settings.

## Conclusions

The present study found that SSI occurred among patients undergoing abdominal surgery. *Escherichia coli *was the predominant pathogen isolated from SSI cases. Most SSI isolates demonstrated antimicrobial susceptibility, although clinical response to broad-spectrum antibiotic therapy varied among patients. Mortality was observed in both elective and emergency surgery groups, but its relationship with SSI was not established. These findings support continued SSI surveillance, early detection, microbiological evaluation, appropriate antimicrobial management, and adherence to infection-prevention measures in abdominal surgical practice.
